# Supplementary material for: Antibiotic-induced DNA damage results in a controlled loss of pH homeostasis and genome instability
Source: Sci Rep. 2020 Nov 10;10:19422. doi: 10.1038/s41598-020-76426-2 (PMC7655802; doi:10.1038/s41598-020-76426-2)
Supplement: Supplementary file 1 — Supplementary Information. [file 41598_2020_76426_MOESM1_ESM.docx]

**Antibiotic-induced DNA damage results in a controlled loss of pH homeostasis and genome instability**

James Alexander Booth,^1,*^ Mário Špírek,^2, 4^ Tekle Airgecho Lobie,^1^ Kirsten Skarstad,^1^ Lumir Krejci,^2,3,4,6^ and Magnar Bjørås^1,5,6,*^

^1^Department of Microbiology, University of Oslo and Oslo University Hospital, Rikshospitalet, Oslo, Norway

^2^Department of Biology, Masaryk University, Kamenice 5/A7, CZ-62500 Brno, Czech Republic

^3^National Centre for Biomolecular Research, Masaryk University, Kamenice 5/A4, CZ-62500 Brno, Czech Republic

^4^International Clinical Research Center, Center for Biomolecular and Cellular Engineering, St. Anne’s University Hospital Brno, Pekarska 53, CZ-656 91 Brno, Czech Republic

^5^Department of Cancer Research and Molecular Medicine, Norwegian University of Science and Technology, Trondheim, Norway.

^6^Contributed equally

^*^Corresponding Authors (james.booth@rr-research.no & magnar.bjoras@rr-research.no)

**Supplementary Results**

**DNA damage leads to the collapse of the electrical potential**

Electrical depolarisation is a hallmark of the SOS response ^1,2^. Our initial step was to confirm previously observed changes in electrical polarity of *E. coli* following DNA damage. To induce DNA damage and the SOS response the bacteria were exposed to the quinolone antibiotic nalidixic acid ^3^. The depolarisation kinetics were similar to those previously reported, an initial delay of 60 minutes is seen before a steady depolarisation over the next 240 minutes (Supplementary Figure S1)^1,2^. The two key upstream regulators in the activation of the SOS response are the single-stranded DNA (ssDNA) binding protein RecA and LexA the RecA-ssDNA nucleoprotein sensitive repressor of the SOS genes. To confirm the SOS response dependency on electrical depolarisation an identical experiment was carried out with a *recA* mutant (Supplementary Figure S1).

**GFP fluorescence is pH dependent**

In order to measure the intracellular pH of bacteria, we used the pH sensitive properties of a fast folding derivative of GFP, GFPmut3* ^4^. Although convenient, observations from GFP based reporter systems should take account of filamentation that occurs during SOS. The fluorescence signal of GFP and other fluorophores have been demonstrated to correlate linearly with cell size and do not necessarily show an increased response due to their underlying inducer ^5,6^. Cell size can be defined by forward scatter (FSC) during flow cytometry, however, side scatter (SSC) has also been shown to be an equally valid measure ^5,6^. Subsequently, observations of fluorophore signal intensities when cell filamentation is observed have used FSC to correct for changes in fluorescence due to cell morphology ^7^. The pH sensitivity of a GFPmut3* containing system was assessed using the membrane permeable weak acid sodium benzoate that rapidly equilibrates the pH across the inner membrane within the permissive range of pHs for neutrophilic *E. coli* 5.5-8 (Supplementary Figure S2). To improve accuracy and to take account of changes in morphology and other possible factors affecting fluorescence a calibration curve was produced for each data point (Supplementary Figure S3). In this example, the calibration curves are biphasic with an increase in fluorescence signal for the first 90 minutes followed by a reduction for the remainder of the time. Together these experiments illustrate how the use of calibration curves for each data point can aid in the production of accurate data.

**Table S1. Strains and plasmids used in this study**

| Strain | Genotype / plasmid | Parent strain | Source |
| --- | --- | --- | --- |
| MG1655 | *F-, λ-, rph-1* | - |  |
| **GFPmut3*** | */ pAra TorA-GFPmut3** | MG1655 | Electroporation of plasmid from ^4^ |
| *ΔrecA* | *ΔrecA* | MG1655 | P1 transduction from JW2669-1 ^8^ and pCP20 removal of Kan^R^ |
| ***recA*** | */ pAra TorA-GFPmut3** | MG1655 *ΔrecA* | Electroporation of plasmid from ^4^ |
| ***recA+*** | */ precA-Htg* | MG1655 *ΔrecA / pAra TorA-GFPmut3** | Electroporation of plasmid from ^9^ |
| *recA-gfp* | *ygaD1::kan recAo1403 recA4136::gfp-901* (*gfp901* refers to *mut-2*) | MG1655 | ^10^, the original genotype SS3041 was from here ^11^ |
| ***p****lexA-gfp* | */ plexApromoter-gfpmut-2* | MG1655 | Electroporation of plasmid from ^12^ |
| ***p****umuDC-gfp* | */ pumuDCpromoter-gfpmut-2* | MG1655 | Electroporation of plasmid from ^12^ |
| *recA K250R* | *recA K250R* | MG1655 | P1 transduction from EAW540† (a generous gift from M. M. Cox) and pCP20 removal of Kan^R^ |
| ***recA K250R GFPmut3**** | */ pAra TorA-GFPmut3** | MG1655 *recA K250R* | Electroporation of plasmid from ^4^ |
| ***recA K250R pumuDC-gfp*** | */ pumuDCpromoter-gfpmut-2* | MG1655 *recA K250R* | Electroporation of plasmid from ^12^ |
| SMR4125 (FC29) | *Δ(lac-proAB)*_XIII_ *ara thi* Rif^S^ *[F′ proAB^+^ Δ(lacI-lacZ)]* | P90C | ^13^ |
| SMR4562 (FC40) | *Δ(lac-proAB)*_XIII_ *ara thi* Rif^R^ *[F′ proAB^+^ lacI33ΩlacZ]* | P90C | ^14^ |

†EAW540 contained an mutant FRT-Kan^R^-wt FRT just after the stop codon of *recX* for transduction and *recA K250R*.

**Figure S1**

**Figure S1. DNA damage results in *recA* dependent depolarisation.**

The polarity of the bacteria was followed using the negatively charge dye DiBAC_4_(3) that increases in fluorescence upon entry into the bacterium. DNA damage was induced in an exponentially expanding population of *E. coli* (MG1655 or MG1655 *recA*) by addition of nalidixic acid (100 µg/ml) at 0 minutes and the fluorescence intensity of DiBAC_4_(3) followed using flow cytometry at the time points indicated. Plotted points are mean ± s.d. (*n* = 3).**Figure S2**

**Figure S2. Fluorescence intensity of intracellular GFPmut3* varies according to pH.**

Calibration of GFPmut3* using buffered media at the pH’s indicated and equilibrated with sodium benzoate in an exponentially expanding suspension of *E. coli*. No DNA damage was induced, fluorescent intensity values were measured by flow cytometry. Plotted points are mean ± s.d. (*n* = 4).**Figure S3.**

**Figure S3. Temporal fluorescence intensity measurement variation following DNA damage results in fluctuations in the calibration curves,**

Calibration of GFPmut3* using buffered media at the pH’s indicated following equilibration with sodium benzoate. DNA damage was induced in an exponentially expanding population by nalidixic acid addition (100 µg/ml) at t=0, fluorescence intensity values were measured by flow cytometry. Plotted points are mean ± s.d. (*n* = 4).**Figure S4.**

**Figure S4. *recA* transcription and cell morphology are dependent on the external pH following DNA damage**

(**A**) The fluorescence signal of *recA-gfpmut2* was followed using flow cytometry.

(**B**) Morphological remodelling was followed by flow cytometry using FSC-A as a proxy for bacterial size.

(**A & B**) The liquid media was buffered to pH 5.2, 6, 7 and 8. DNA damage (nalidixic acid 100 µg/ml) was induced at t=0 min and error bars are mean ± s.e.m., *n* =4 (independent replicates).**Figure S5.**

**Figure S5. *umuDC* transcription and cell morphology are dependent on the external pH following DNA damage**

(**A**) The fluorescence signal of *pumuDC-gfpmut2* was followed using flow cytometry. (**B**) Morphological remodelling was followed by flow cytometry using FSC-A as a proxy for bacterial size.

(**A & B**) The liquid media was buffered to pH 5.2, 6, 7 and 8. DNA damage (nalidixic acid 100 µg/ml) was induced at t=0 min and error bars are mean ± s.e.m., *n* =4 (independent replicates).**Figure S6.**

**Figure S6. The effect of pH on binding of ssDNA and recombinase activity**

(**A**) Gel illustrating the effect of pH on RecA DNA binding activity. Purified RecA protein was incubated with 90mer FITC-labelled oligonucleotide (10 nM) in the presence of 1 mM ATP and 10 mM MgCl_2_ for 5 minutes at RT, followed by addition of glutaraldehyde to final concentration 0.125%. Protein-DNA complexes were resolved on 1% agarose gel. RecA monomer versus DNA nucleotide ratio is indicated. Enumeration of the band intensities are shown in Figure 2A. p.28 contains the raw image file.

(**B**) Indicated amounts of RecA protein preincubated with ATP were rapidly mixed with 20 nM 5´Cy3-labelled dT79 oligonucleotide in the presence of 1 mM ATP and 10 mM MgCl_2_ in stop-flow set-up at various pH conditions. Change of fluorescence was observed from time 0 to 120 sec.

(**C**) Gels illustrating the effect of pH on RecA-mediated D-loop formation. Increasing amounts of RecA protein (0.4, 0.7, 1, 1.5, 2.3, 3.4 and 5.1 μM) were pre-mixed with 50 nM FITC labelled 90mer ssDNA followed by addition of dsDNA plasmid to yield D-loop structure. All samples were then treated with SDS and proteinase K (SDS/PK) to release the DNA substrates and analysed by PAGE. p.29 contains the raw image file.

**Figure S7.**


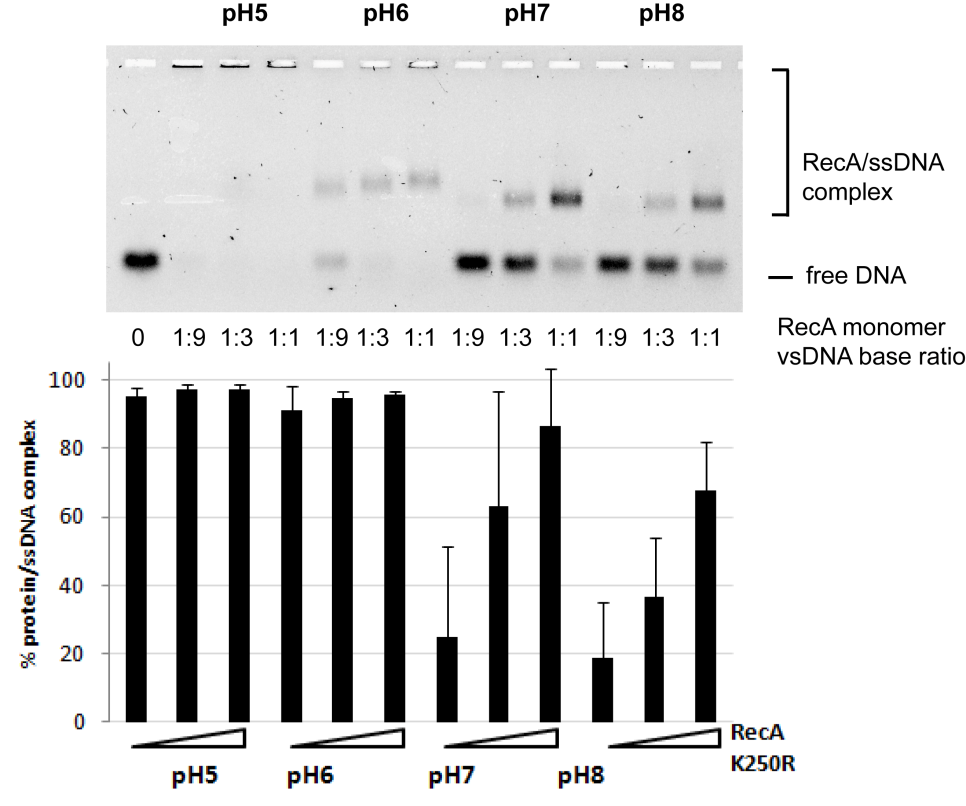


**Figure S7. pH dependence of RecA K250R-ssDNA binding.**

Gel illustrating the effect of pH on RecA K250R DNA binding activity. Purified RecA K250R protein was incubated with 90mer FITC-labelled oligonucleotide (10 nM) in the presence of 1 mM ATP and 10 mM MgCl_2_ for 5 minutes at RT, followed by addition of glutaraldehyde to final concentration 0.125%. Protein-DNA complexes were resolved on 1% agarose gel. RecA monomer versus DNA nucleotide ratio is indicated (mean ± s.d. error bars, (*n* = 3)). p.29 contains the raw images file.

**Figure S8.**

**Figure S8. External pH dependence of the MIC of various classes of antibiotics**

A seed culture of *E. coli* was added to media containing various concentrations of antibiotics (**A**. nalidixic acid, quinolone, **B**. norfloxacin, fluoroquinolone, **C**. ampicillin, beta-lactam, **D**. rifampicin, polyketide/ansamycin, **E**. zeocin, glycopeptides/phleomycin, **F** & **G**. streptomycin and kanamycin respectively, aminoglycosides, **H**, erythromycin, macrolide. After overnight incubation the bacterial density was determined by optical density measurements (OD_595_). All antibiotics showed varying potency over the range of pH’s used, however, the range of potency was highest for those containing sugar moieties.

**Figure S9**

**Figure S9. The inherent variation in colony based survival assays made the significance of external pH hard to determine.**

Colony based survival assay of exponentially growth cultures of MG1655 (**A**) and MG1655 *recA* (**B**) following exposure to nalidixic acid 100 µg/ml at time point 0 min at the pH’s indicated. Samples removed at the time points indicated were subsequently plated on LB to determine viable colony counts. Data points are mean±s.e.m error bars (n=3 independent replicates).

**Raw Image files**

**
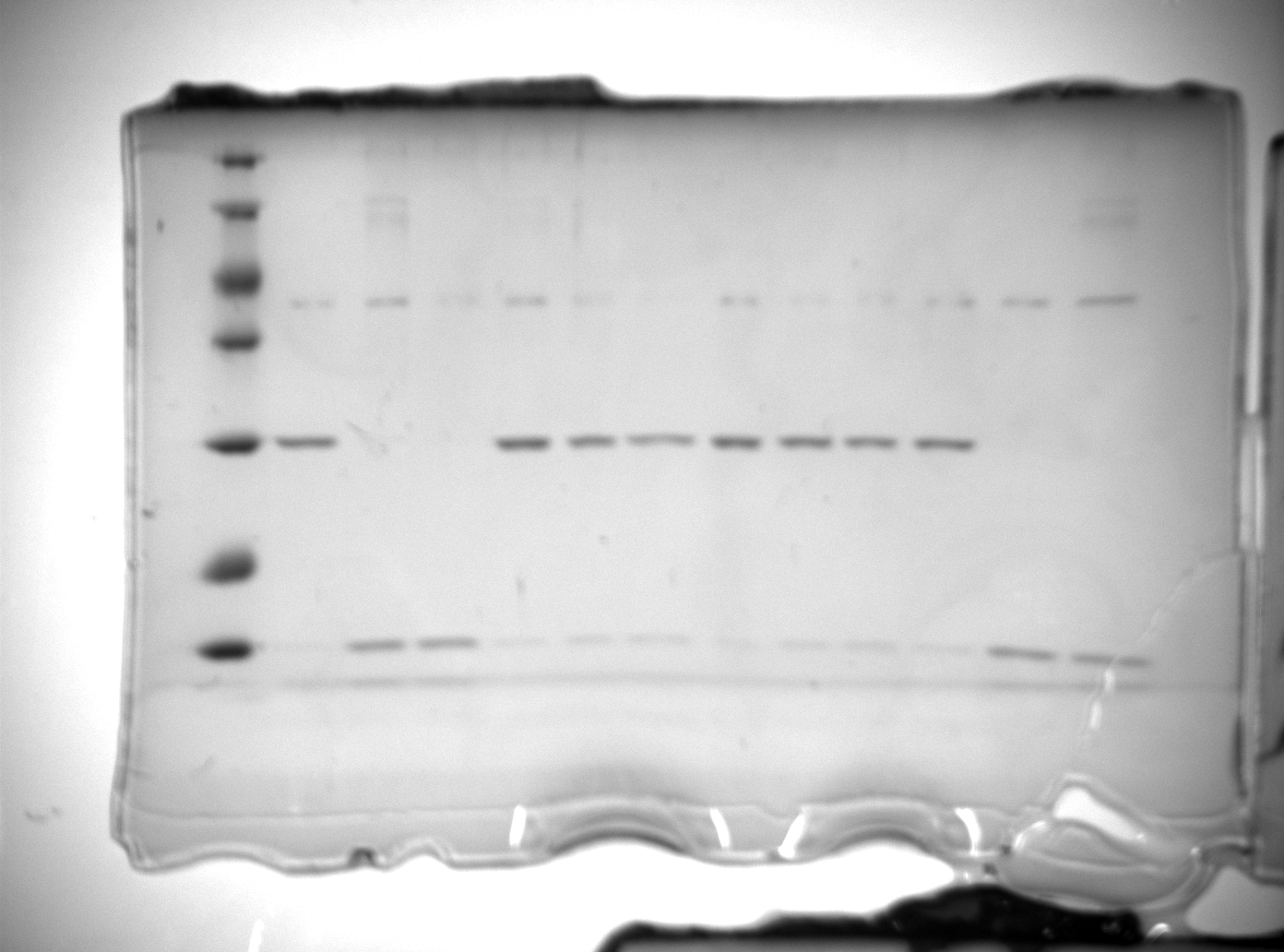
**

**Fig 5A.**

**
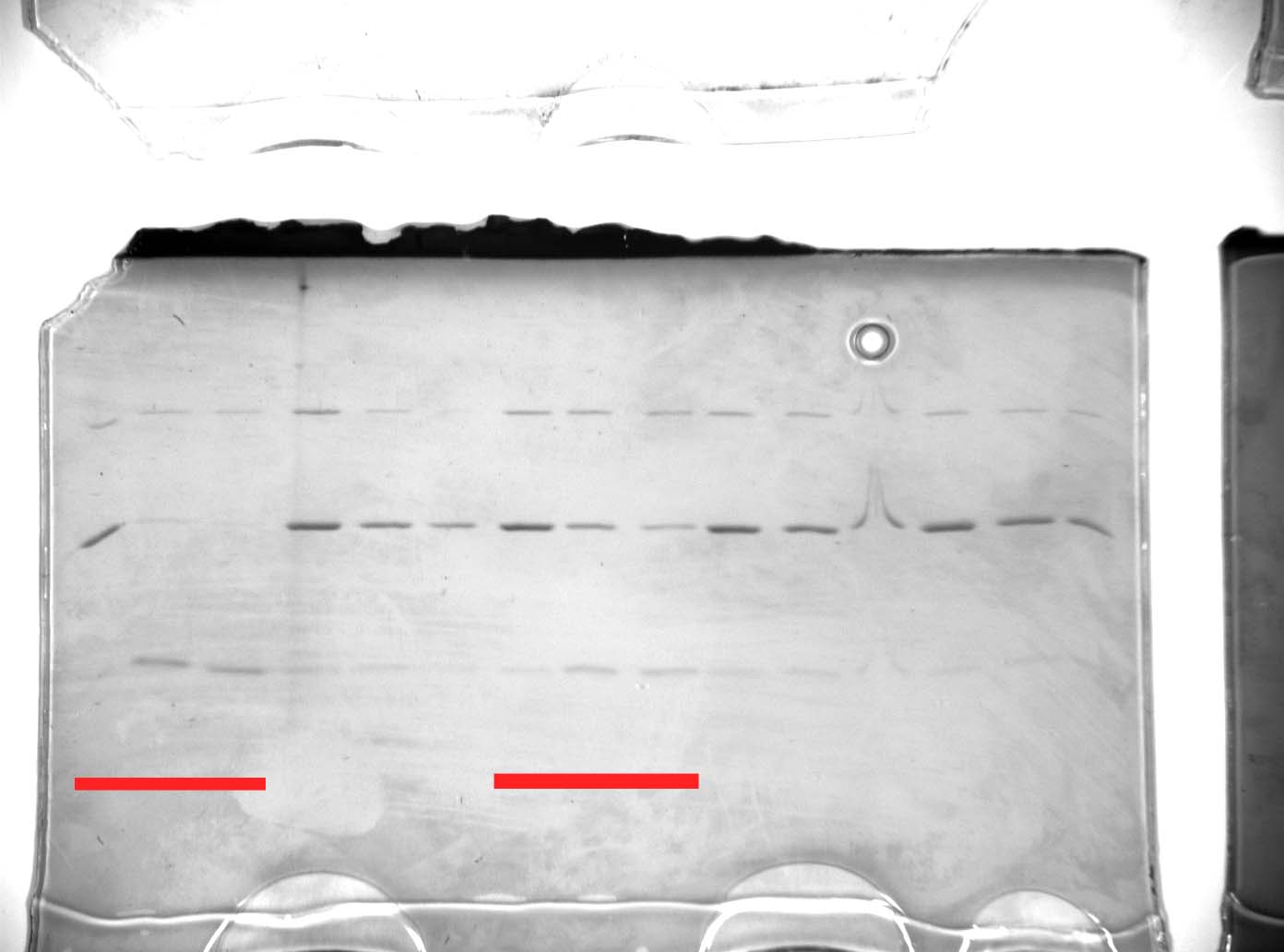
**

**Fig 5B.**

**
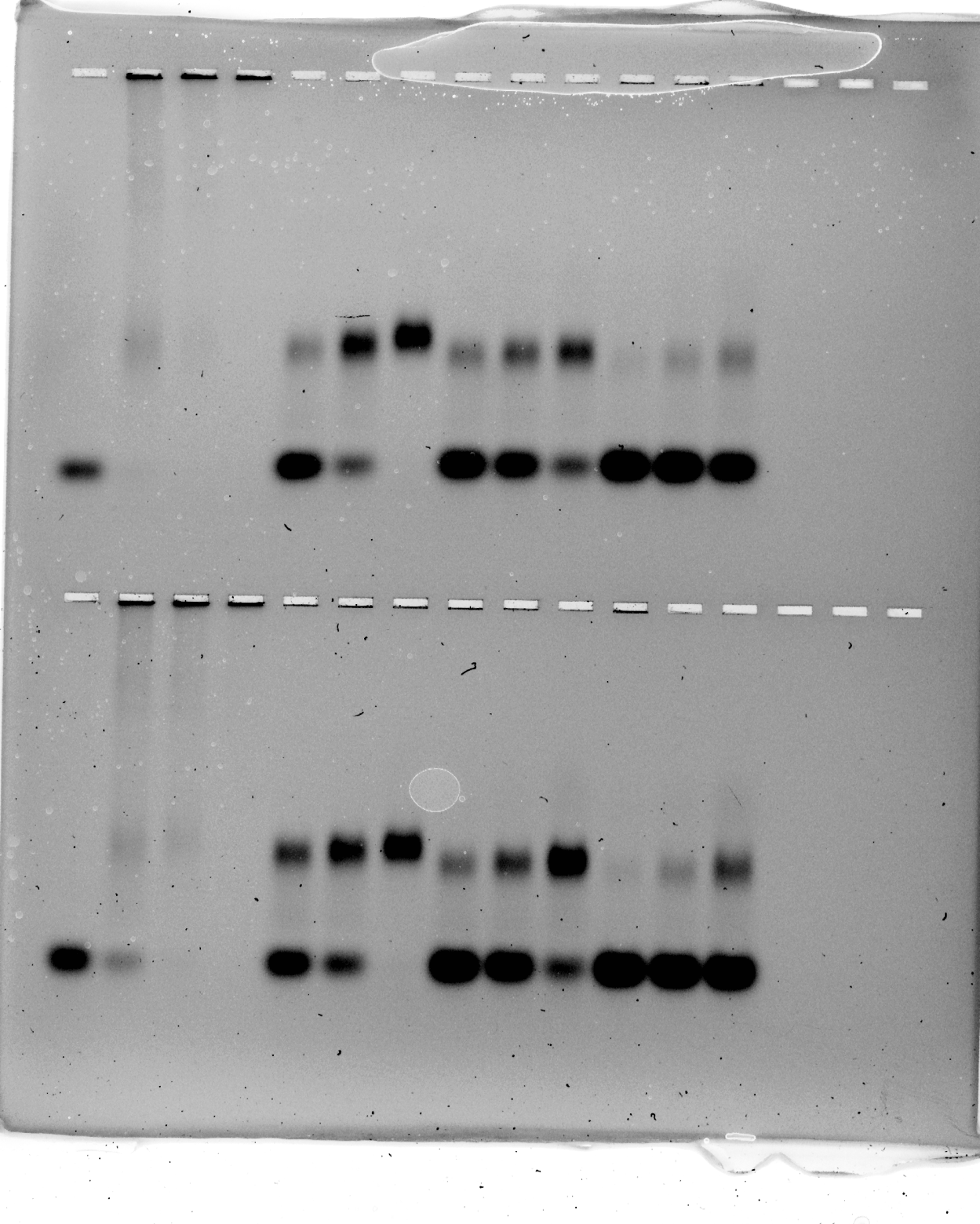
**

**Fig S6A**

**
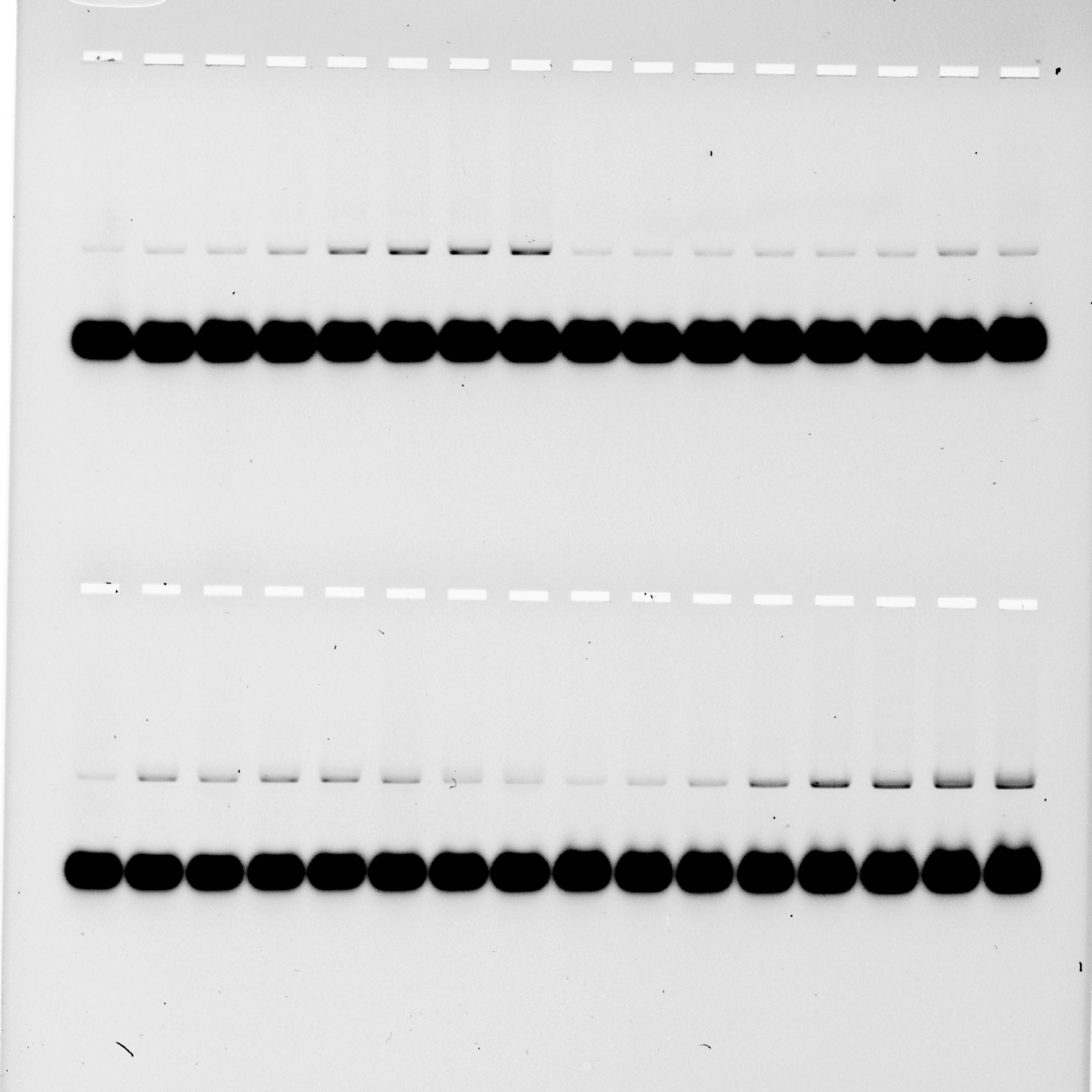
**

**Fig S6C.**

**
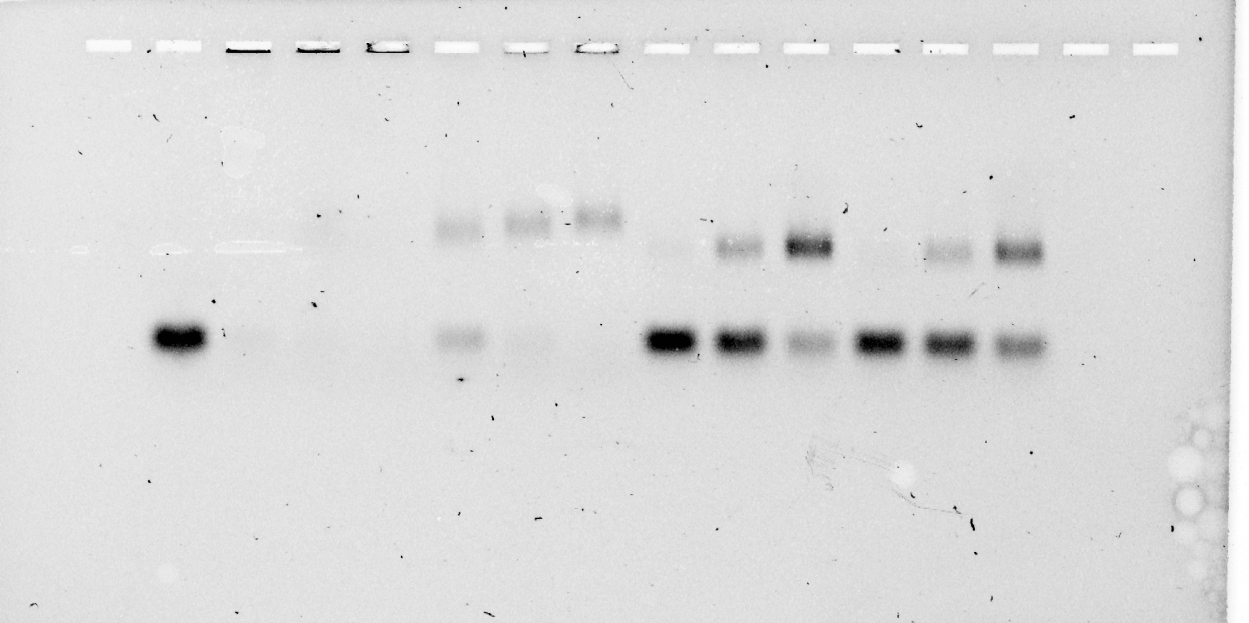
**

**Fig S7**

**References**

1 Erental, A., Sharon, I. & Engelberg-Kulka, H. Two programmed cell death systems in Escherichia coli: an apoptotic-like death is inhibited by the mazEF-mediated death pathway. *PLoS Biol* **10**, e1001281 (2012).

2 Dwyer, D. J., Camacho, D. M., Kohanski, M. A., Callura, J. M. & Collins, J. J. Antibiotic-induced bacterial cell death exhibits physiological and biochemical hallmarks of apoptosis. *Mol Cell* **46**, 561-572 (2012).

3 Drlica, K. & Zhao, X. L. DNA gyrase, topoisomerase IV, and the 4-quinolones. *Microbiology and Molecular Biology Reviews* **61**, 377-+ (1997).

4 Wilks, J. C. & Slonczewski, J. L. pH of the cytoplasm and periplasm of Escherichia coli: rapid measurement by green fluorescent protein fluorimetry. *J Bacteriol* **189**, 5601-5607 (2007).

5 Paulander, W. *et al.* Bactericidal Antibiotics Increase Hydroxyphenyl Fluorescein Signal by Altering Cell Morphology. *Plos One* **9**; e92231 (2014).

6 Renggli, S., Keck, W., Jenal, U. & Ritz, D. Role of Autofluorescence in Flow Cytometric Analysis of Escherichia coli Treated with Bactericidal Antibiotics. *Journal of Bacteriology* **195**, 4067-4073 (2013).

7 Dewachter, L., Herpels, P., Verstraeten, N., Fauvart, M. & Michiels, J. Reactive oxygen species do not contribute to ObgE(star)-mediated programmed cell death. *Sci Rep-Uk* **6**; 33723 (2016).

8 Baba, T. *et al.* Construction of Escherichia coli K-12 in-frame, single-gene knockout mutants: the Keio collection. *Mol Syst Biol* **2**, 2006.0008. (2006).

9 Kitagawa, M. *et al.* Complete set of ORF clones of Escherichia coli ASKA library (A complete Set of E. coli K-12 ORF archive): Unique resources for biological research. *DNA Res* **12**, 291-299 (2005).

10 Rajendram, M. *et al.* Anionic Phospholipids Stabilize RecA Filament Bundles in Escherichia coli. *Molecular Cell* **60**, 374-384 (2015).

11 Renzette, N. *et al.* Localization of RecA in Escherichia coli K-12 using RecA-GFP. *Mol Microbiol* **57**, 1074-1085 (2005).

12 Friedman, N., Vardi, S., Ronen, M., Alon, U. & Stavans, J. Precise temporal modulation in the response of the SOS DNA repair network in individual bacteria. *PLoS Biol* **3**, e238 (2005).

13 Cairns, J. & Foster, P. L. Adaptive Reversion of a Frameshift Mutation in Escherichia-Coli. *Genetics* **128**, 695-701 (1991).

14 McKenzie, G. J., Harris, R. S., Lee, P. L. & Rosenberg, S. M. The SOS response regulates adaptive mutation. *Proceedings of the National Academy of Sciences of the United States of America* **97**, 6646-6651 (2000).
